# Sulfate-Reducing Naphthalene Degraders Are Picky Eaters

**DOI:** 10.3390/microorganisms6030059

**Published:** 2018-06-25

**Authors:** Sarah J. Wolfson, Abigail W. Porter, Lee J. Kerkhof, Lora M. McGuinness, Roger C. Prince, Lily Y. Young

**Affiliations:** 1 Department of Environmental Sciences, Rutgers University, New Brunswick, NJ 08901, USA; sarah.wolfson@rutgers.edu (S.J.W.); abigail.porter@rutgers.edu (A.W.P.); 2 Department of Marine and Coastal Sciences, Rutgers University, New Brunswick, NJ 08901, USA; lkerkhof@rutgers.edu (L.J.K.); mcguinness@marine.rutgers.edu (L.M.M.); 3 Stonybrook Apiary, Pittstown, NJ 08867, USA; rogercprince@gmail.com

**Keywords:** naphthalene, anaerobic, biodegradation

## Abstract

Polycyclic aromatic hydrocarbons (PAHs) are common organic contaminants found in anoxic environments. The capacity for PAH biodegradation in unimpacted environments, however, has been understudied. Here we investigate the enrichment, selection, and sustainability of a microbial community from a pristine environment on naphthalene as the only amended carbon source. Pristine coastal sediments were obtained from the Jacques Cousteau National Estuarine Research Reserve in Tuckerton, New Jersey, an ecological reserve which has no direct input or source of hydrocarbons. After an initial exposure to naphthalene, primary anaerobic transfer cultures completely degraded 500 µM naphthalene within 139 days. Subsequent transfer cultures mineralized naphthalene within 21 days with stoichiometric sulfate loss. Enriched cultures efficiently utilized only naphthalene and 2-methylnaphthalene from the hydrocarbon mixtures in crude oil. To determine the microorganisms responsible for naphthalene degradation, stable isotope probing was utilized on cultures amended with fully labeled ^13^C-naphthalene as substrate. Three organisms were found to unambiguously synthesize ^13^C-DNA from ^13^C-naphthalene within 7 days. Phylogenetic analysis revealed that 16S rRNA genes from two of these organisms are closely related to the known naphthalene degrading isolates NaphS2 and NaphS3 from PAH-contaminated sites. A third 16S rRNA gene was only distantly related to its closest relative and may represent a novel naphthalene degrading microbe from this environment.

## 1. Introduction

Polycyclic aromatic hydrocarbons (PAHs) are ubiquitous in the environment from biogenic, pyrogenic, and more recently, anthropogenic sources. Increases in anthropogenic production have led to areas with substantial concentrations, even in pristine environments historically un-impacted by point source pollution [1]. These toxic chemicals are of concern because of commonplace, continual anthropogenic introduction. 

Of the major PAH fate pathways in sediment, including volatilization, adsorption, and chemical degradation, microbial mineralization is the most important degradation process [2]. Aerobic PAH biodegradation is well characterized. PAHs, however, quickly enter anoxic environments via sorption to organic particulates in the water column and subsequent burial in the sediment [3]. Once PAHs enter anoxic regions of soils and sediments, anaerobic processes become important. Naphthalene has been shown to be metabolized under sulfate-reducing conditions [3]. Two sulfate-reducing bacterial strains capable of naphthalene degradation, NaphS2 and NaphS3, have been isolated from heavily polluted marine sediment in the Etang de Berre, a Mediterranean lagoon, and the North Sea [4,5]. Sulfate reducers from freshwater aquifers contaminated with hydrocarbon have also been described, including strain N47 [6,7]. These strains all belong to the family *Desulfobacteraceae*. In addition to consuming naphthalene, the characterized sulfate-reducing naphthalene degraders also metabolize 2-methylnaphthalene [8]. Crude oil, however, is a mixture of different chemicals and the metabolic diversity of anaerobic naphthalene degraders with this carbon source has not been reported. This is of interest since naphthalene is usually found along with other petroleum constituents in the environment. 

Investigations of PAH-degrading microbes have focused on sites with known hydrocarbon contamination from industrial use or oil spills (Table 1). In this study, however, we assessed anaerobic naphthalene degradation by microbes from sediment unimpacted by large-scale hydrocarbon contamination. Samples were obtained from the Jacques Cousteau National Estuarine Research Reserve along the New Jersey (USA) seaboard, which has no natural oil seeps and no history of substantial hydrocarbon contamination [9]. Nevertheless, this pristine site, like many environments, receives low levels of PAHs from non-point sources [1]. Here, we report on specific microbes from this site capable of degrading naphthalene under sulfate-reducing conditions. These naphthalene biodegraders include a new bacterial taxon, expanding the breadth of known naphthalene-degrading bacteria in marine ecosystems. 

## 2. Materials and Methods 

### 2.1. Chemicals

Chemicals were purchased from Sigma-Aldrich (St. Louis, MO, USA) and Thermo Scientific (Waltham, MA, USA). Fully labeled ^13^C_10_-naphthalene with chemical and isotopic purity of 99% was purchased from Sigma-Aldrich. 

### 2.2. Site Description

Enrichment cultures were established using sediment from the Jacques Cousteau National Estuarine Research Reserve, a protected marine salt marsh in Tuckerton, NJ. The concentration of total PAHs in this site is lower than total PAHs in inoculum of previous naphthalene degrading studies (Table 1). Anoxic subsurface sediment was used as inoculum for naphthalene degrading microcosms. This estuarine system is highly productive and as a result, the sediment is highly reducing. Sediment was maintained under an N_2_ atmosphere at 4 °C for 24 h before enrichment cultures were established.

### 2.3. Enrichment Cultivation

A 20% (*v*/*v*) sediment slurry was added to anaerobic sulfate-reducing marine medium, as described previously [3,15]. ^12^C-naphthalene was the sole added carbon source to the primary enrichment cultures. To deliver the substrate to cultures, naphthalene was dissolved in hexane and added to each bottle. Hexane was evaporated, leaving naphthalene coating the surface of glass serum bottles. The final concentration of naphthalene was 500 μM in 100 mL of the sediment slurry. Cultures were maintained under an N_2_/CO_2_ atmosphere (70:30). Bottles were sealed with butyl rubber stoppers and aluminum crimps. Enrichments were established in triplicate, and duplicate heat-killed sterile controls were autoclaved three times on three consecutive days. All bottles were incubated at room temperature in the dark. 

Primary enrichment cultures were transferred after one year of incubation. Transfers were established with 3% primary culture in 97% sulfate-reducing marine media, which contained 20 mM sulfate [15]. The first transfer received naphthalene as described above, for a final concentration of 500 µM in each culture. To facilitate naphthalene re-amendment, subsequent naphthalene amendments were provided via silica carrier. Naphthalene dissolved in hexane was mixed with silica particles, and then hexane was evaporated, producing a naphthalene-sorbed silica stock of a calculated concentration of 50 µmol per gram silica. A small portion of the naphthalene was expected to volatilize during solvent evaporation. Following the initial 3% transfer, subsequent transfer cultures received 50% culture and 50% fresh media. These consortia were amended with enough naphthalene-sorbed silica to provide a final concentration of 500 μM naphthalene. Cultures were amended three times before transfer. Complete naphthalene disappearance was confirmed before each re-amendment. To determine the effect of inhibiting sulfate reduction, two subcultures received molybdate (MoO_4_^2−^) at 20 mM [3]. 

### 2.4. Oil Biodegradation

Subcultures of sediment-free enrichments were amended with crude oils to determine the range of petroleum hydrocarbons used as substrates by the enriched culture [16]. Artificially weathered Alaska North Slope crude oil (API = 29°), depleted in monoaromatic compounds and low molecular weight alkanes, was amended at 5 µL/30 mL culture. Alba crude oil (API = 19.4°) was amended to a separate set of subcultures at 5 µL/30 mL culture. This crude oil was not weathered and contains monoaromatic constituents. Inactive controls of each treatment received molybdate to inhibit sulfate reduction. Entire bottles of triplicate active and duplicate molybdate inactivated cultures were sacrificed and stored frozen at each time point for hydrocarbon analysis. 

### 2.5. Chemical Analysis

Naphthalene concentration was monitored using a Hewlett-Packard 5890 Series II gas chromatograph with a flame ionization detector (Hewlett-Packard, Palo Alto, CA, USA) and a 30 m × 0.32 mm DB-WAX capillary column (J & W Scientific, Folsom, CA, USA). To quantify naphthalene in each culture, 0.75 mL samples were mixed with an equal volume of hexane containing 500 µM fluorene as an internal standard. Naphthalene was detectable below 62.5 µM. Sulfate was measured using a Dionex model DX-120 ion chromatograph (Sunnyvale, CA, USA) equipped with an IonPac AS9 column and conductivity detector. Eluent was composed of 2 mM Na_2_CO_3_ and 0.75 mM NaHCO_3_ at 1 mL min^−1^. Total hydrocarbon loss in crude oil subcultures was monitored using a Hewlett-Packard HP-5890 GC/MS. Thawed bottles were extracted with methylene chloride and processed as described previously [16]. 

### 2.6. Stable Isotope Probing (SIP) Microcosms

A sediment-free ^12^C-naphthalene degrading consortium was transferred into 50% fresh media and amended with uniformly labeled ^13^C_10_-naphthalene as the sole carbon source at a final concentration of 390 µM. Parallel cultures were amended with ^12^C-naphthalene. Subsamples (1 mL) from the SIP cultures and ^12^C bottles were collected every 2–3 days for 17 days for DNA analysis. 

### 2.7. SIP Molecular Analysis

The 1 mL of subsample was filtered onto a 13 mm diameter 0.22 µm filter and immediately frozen until DNA extraction. Genomic DNA was purified using a modified phenol-chloroform procedure with the addition of ^12^C-labeled and ^13^C-labeled archaeal carrier DNA from *Halobacterium salinarum*. ^12^C-DNA and ^13^C-DNA were separated by CsCl density gradient centrifugation at 250,000× *g* [17]. Therefore, each cesium gradient would contain both a ^12^C and a ^13^C band containing both bacterial and archaeal DNA. The bacterial 16S rRNA genes in the ^12^C and ^13^C DNA bands were analyzed by PCR amplification with bacterial-specific primers 27F (5′-AGAGTTTGATCMT GGCTCAG-3′), labeled with a 6-carboxyfluorescein at the 5′ end, and 1100R (5′-GGGTTGCGCTCGTTG-3′). These PCR amplicons (20 ng DNA) were digested using *Mnl1* endonuclease (New England Biolabs, Beverly, MA, USA) for 6 h at 37 °C and analyzed by T-RFLP using an ABI 310 Genetic Analyzer (Applied Biosystems, Foster City, CA, USA). The relative abundance of each identified TRF peak was calculated by normalizing its peak area to the total area of the entire profile as described previously [18]. 

Ribosomal RNA genes corresponding to the terminal restriction fragments (TRFs) in the SIP analysis were identified from a clone library of ^13^C amplicons collected at day 7 using a pGEM cloning kit and chemically transformed into *E. coli* JM109 competent cells (Promega, Madison, WI, USA) according to the manufacturer’s instructions. 16S rRNA gene PCR products were sequenced by Genewiz (Genewiz Inc., South Plainfield, NJ, USA) using T7F and M13R primers. Taxonomic identity was assigned by comparing cloned sequences with the NCBI nucleotide collection nr/nt database using BlastN [19]. Sequences were deposited to GenBank under accession numbers MH446976–MH446979. 

## 3. Results and Discussion

### 3.1. Naphthalene Degrading Enrichments

**Figure 1:**
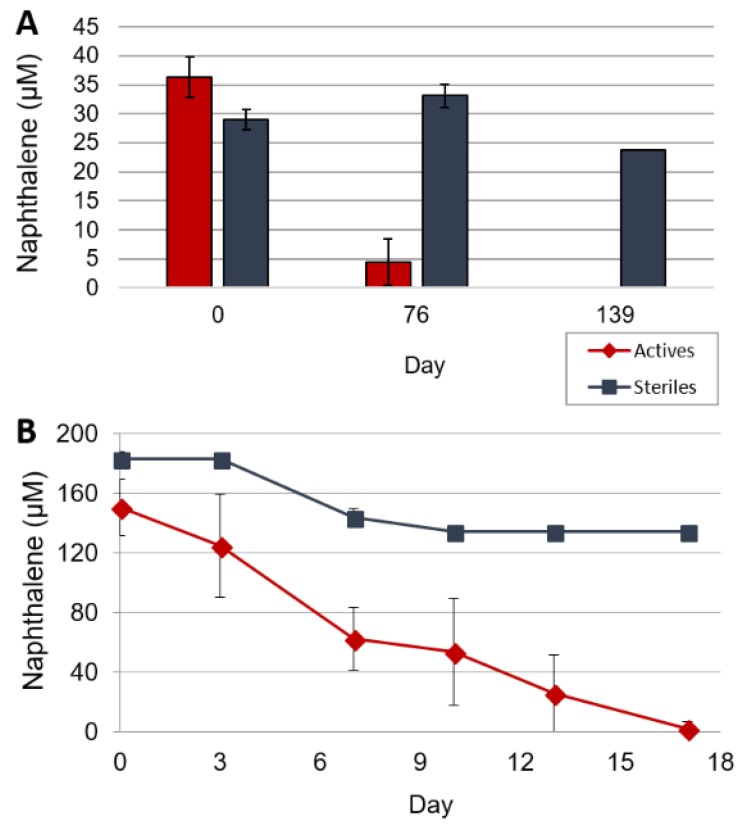
Average naphthalene loss in anaerobic cultures and sterile controls. (**A**) Primary Transfer Cultures, naphthalene delivered by sorption to glass culture vessels; (**B**) Enriched Cultures, naphthalene delivered by sorption to silica. Data are averages of triplicate active cultures and duplicate sterile controls. (♦) Active Cultures (■) Sterile Controls. Error bars represent standard deviation between replicate cultures. Error bars are present for all data points, if they are not visible they are within the marker size.

Naphthalene was no longer detected in primary enrichment cultures after one year. The first transfer cultures completely degraded naphthalene in 139 days (Figure 1A) with limited naphthalene loss seen in sterile controls. Naphthalene recovery in primary enrichment cultures was only 10%, as naphthalene had been delivered by coating the glass culture bottles and would have to desorb from the glass bottles into the aqueous medium. After naphthalene was degraded by primary enrichments, the cultures were transferred to fresh media and silica was used to deliver naphthalene. The naphthalene-sorbed silica provided increased surface area and allowed for a homogenous dispersal throughout the liquid culture. Silica delivery also improved recovery to 40%, which is typical for PAH biodegradation assays [3]. Subsequent naphthalene amendments and transfers resulted in a sediment-free enriched consortium capable of degrading 500 µM naphthalene in 17 days (Figure 1B). 

**Figure 2:**
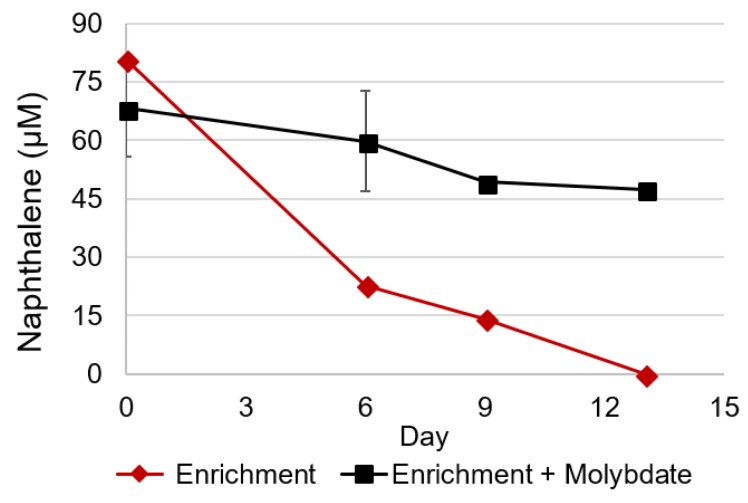
Naphthalene loss with and without molybdate amendment. (♦) Enrichment Cultures (■) Enrichment Cultures with Molybdate Amendment. Error bars represent standard deviation between triplicate cultures. Error bars are present for all data points. If they are not visible they are within the marker size.

To determine whether naphthalene degradation was coupled to sulfate reduction, the electron acceptor concentration was monitored in primary transfer cultures. Due to the long primary enrichment incubation and low (3%) transfer volume, little background carbon is likely to have been present. Thus, all sulfate loss was expected to be as a result of naphthalene metabolism. Three mM sulfate loss was observed in primary transfers, which received 500 μM naphthalene (Table 2). This loss is consistent with the predicted electron acceptor loss based on stoichiometric values (Table 2). Furthermore, when sulfate reduction was inhibited by the addition of molybdate, little naphthalene loss was observed (Figure 2) [3]. In the absence of molybdate, however, naphthalene was metabolized within 13 days. Thus, naphthalene metabolism is directly linked to sulfate reduction in this consortium. 

### 3.2. Crude Oil Biodegradation

**Figure 3:**
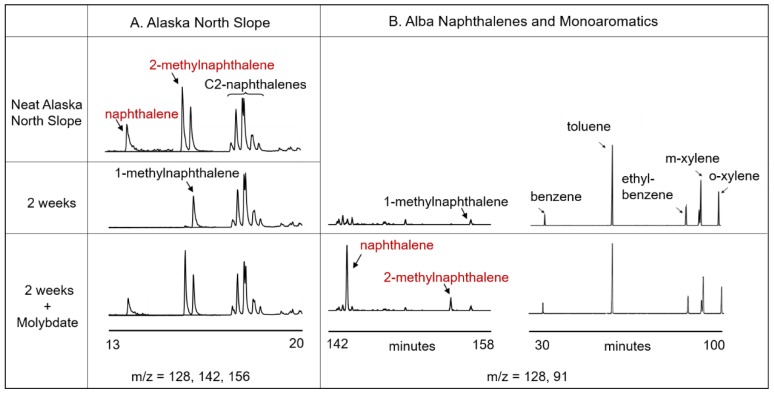
Selective naphthalene degradation in (**A**) Alaska North Slope and (**B**) Alba crude oils. Top row, crude oil. Middle row, the profile of cultures following a 2-week incubation. Bottom row, 2-week incubation of cultures amended with molybdate to inhibit respiration. Naphthalene and 2-methylnaphthalene are indicated in red. Unmetabolized aromatics are indicated in black. The m/z of 128, 142, and 156 are combined.

As shown in Figure 3A, naphthalene degrading cultures amended with weathered Alaska North Slope crude oil, which is rich in PAHs, readily degraded naphthalene and 2-methylnaphthalene. The two compounds were completely degraded within two weeks in active cultures, while no loss was observed in cultures inhibited with molybdate (Figure 3A middle and bottom rows). Anaerobic naphthalene degrading bacteria are known to metabolize 2-methylnaphthalene [3,8,20]. Sullivan et al. [8] demonstrated that 2-methylnaphthalene shares the same degradation pathway as naphthalene once both compounds are carboxylated to 2-naphthoic acid. Moreover, both compounds share the same five intermediates that follow 2-naphthoic acid before ring reduction and cleavage [3,8]. Our results on the degradation of both naphthalene and 2-methylnaphthalene (Figure 3A,B), therefore, underscore the reported anaerobic naphthalene degradation mechanism. This culture is unable to metabolize 1-methylnaphthalene, which is also consistent with observations from other sulfate-reducing communities [21]. After an additional 14 weeks of incubation, no loss of any components besides naphthalene and 2-methylnaphthalene was observed. 

Cultures were also incubated with Alba crude oil to examine alkane and monoaromatic degradation in addition to PAHs. As shown in Figure 3B, during incubation with Alba crude oil, monoaromatic compounds were not degraded, even when naphthalene and 2-methylnaphthalene loss occurred. In these cultures, benzene, toluene, and xylenes persisted. Additionally, 1-methylnaphthalene, PAHs, and alkanes all remained. Recovery of these volatile constituents confirmed that volatilization loss did not occur, and that naphthalene and 2-methylnaphthalene were the only compounds used as a metabolic substrate by this highly selective consortium. 

It is noteworthy and unexpected that despite degrading 2-methylnaphthalene, cultures amended with Alba crude oil showed no toluene or alkane loss over the 16-week experiment (Figure 3). 2-methylnaphthalene, toluene, and alkanes are activated via fumarate addition to the methyl group, suggesting that the mechanism that allows the consortium to degrade 2-methylnaphthalene would also enable toluene metabolism. The enzymes show clear substrate-specificity, however, and it is possible that given more time the microbes would begin toluene degradation [5,22]. Thus, the microbial community in this consortium is highly specialized for naphthalene degradation. This is in contrast to the sulfate reducer NaphS2, which does degrade both toluene and 2-methylnaphthalene [23]. 

### 3.3. Stable Isotope Probing

To determine which microorganisms in the enrichments were capable of naphthalene biodegradation, we performed a SIP experiment on subcultures using ^13^C_10_-naphthalene. Control experiments using ^12^C-naphthalene were also established. All SIP gradients contained both a ^12^C band and a ^13^C carrier bands as described above. To demonstrate unambiguous ^13^C-DNA synthesis, we expected no bacterial 16S rRNA genes amplified in the ^13^C carrier band in the control culture amended with ^12^C-naphthalene. A distinct amplicon should be detected in the ^13^C carrier band when the microcosms were amended with ^13^C-naphthalene. A minimum incubation time of 7 days was required to observe a faint but distinct 16S rRNA gene amplicon in the ^13^C carrier band that received the ^13^C-naphthalene amendment (Supplemental Figure S1); under the same conditions there was no amplification in the ^13^C carrier band with ^12^C-naphthalene (Supplemental Figure S1 band 3). TRFLP profiling of 16S rRNA gene amplicons from ^13^C-DNA was compared at days 7, 14, and 17 of incubation to visualize changes in the microbial community as organisms degrade ^13^C-naphthalene and incorporate the ^13^C-label into DNA. These time points are related to the typical rate of naphthalene degradation in sulfidogenic cultures (Figure 1B).

**Figure 4:**
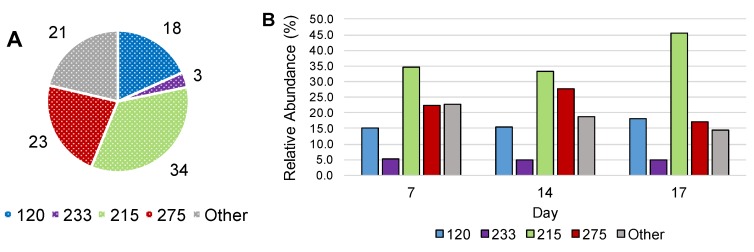
Community profile of naphthalene-degrading enrichment culture. (**A**) Relative abundance as a percent of total identified TRF (120, 233, 215, and 275) and all other combined unidentified peaks before incubation with ^13^C-naphthalene; (**B**) Relative abundance of each identified TRF following incorporation of ^13^C during SIP incubation on days 7, 14, and 17.

TRFLP-16S rRNA gene profiling of the naphthalene degrading microcosm at Day 0 of the SIP experiment, composed entirely of ^12^C-DNA, indicated TRFs 120, 215, 233, and 275 as the most abundant members of the community (Figure 4A). Figure 4B illustrates the relative abundance of these TRFs in the ^13^C-DNA fractions at days 7, 14, and 17. All TRFs incorporated ^13^C from ^13^C-naphthalene at the earliest time point taken during the SIP experiment. TRFs 120 and 233 either showed slight increases at the end of the SIP incubation or largely retained in the same relative abundance throughout the SIP incubation. The most immediate change in relative proportion was for TRF 275, which increased from 22 to 28% of the community by day 14. Notably, the relative abundance of TRF 275 was highest at day 14, concurrent with the greatest extent of naphthalene consumption (Figure 1B). TRF 215 also demonstrated a delayed increase in relative abundance, suggesting consumption of the metabolic by-products generated by the primary naphthalene-degrading members of the community. Specifically, TRF 215 which was present at 34% of the community at days 7 and 14, increased to 46% between days 14 and 17. This increase in relative abundance occurred after naphthalene was almost completely exhausted in cultures (Figure 1B). The data demonstrate the microbial community incorporating ^13^C-naphthalene is relatively stable over time, which was expected for a highly enriched consortium that had been receiving naphthalene as the sole carbon source. These results indicate that the majority of the community is directly involved in naphthalene cycling or consuming the metabolites of biodegradation. 

Identification of the 16S rRNA genes associated with the four TRFs prominent in the ^13^C fraction was accomplished by screening a clone library made from the day 7 ^13^C-DNA fraction. Analysis of 130 clones yielded 1100 bp fragments of 16S rRNA genes corresponding with TRFs 120, 215, 233, and 275. The four TRFs represented 80% of the ^13^C-labeled community. The 16S rRNA genes corresponding to TRF 120 and 233 shared 100% and 99% identity, respectively, to NaphS2 (AJ132804) and NaphS3 (EU908726), which are known anaerobic naphthalene degraders in the family *Desulfobacteraceae*. TRF 215 was identified as a member of the genus *Prosthecochloris*, sharing 99% sequence identity with strains of *P. vibrioformis*. The 16S rRNA gene sequence of TRF 275 was not closely related to any cultured organism, sharing only 84% identity with species in the genera *Desulfomicrobium* and *Desulfocaldus*.

**Figure 5:**
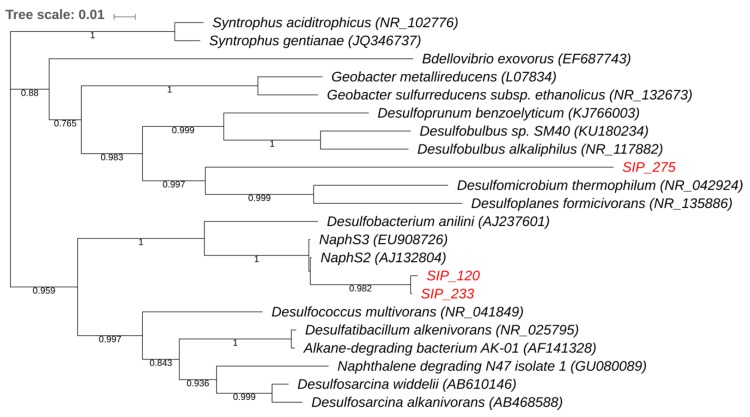
Phylogeny of TRFs 120, 233, and 275. Partial sequence of the 16S rRNA gene of clones with sequences demonstrated to incorporate labeled naphthalene are reported. TRFs of these clones are indicated in red, and their closest matches in Genbank as well as additional reference sequences are included. Sequences were aligned using MUSCLE 3.8.31 [24], constructed by FastTree 2.1.8 [25], and visualized in ITOL [26]. Bootstrap values based on 1,000 iterations are reported on the branches. Only values greater than 0.5 are shown.

**Figure 6:**
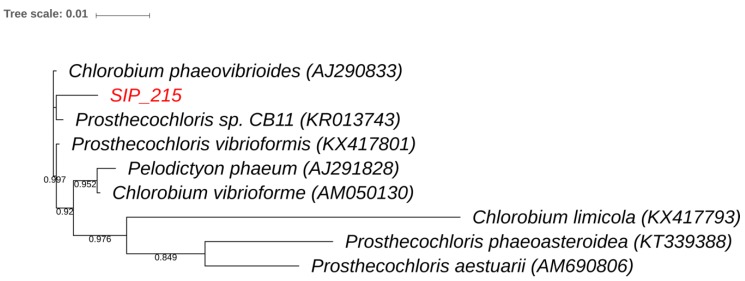
Phylogeny of TRF 215. Partial sequence of the 16S rRNA gene of clones with sequences demonstrated to incorporate labeled naphthalene are reported. The closest matches in Genbank as well as additional reference sequences are included. Sequences were aligned using MUSCLE [24], constructed by FastTree [25], and visualized in ITOL [26]. Bootstrap values based on 1,000 iterations are reported on the branches. Only values greater than 0.5 are shown.

Phylogenetic analysis of the sequenced clones and their closest BLAST matches are displayed in Figure 5 and Figure 6. The *Proteobacteria* tree (Figure 5) shows that the 16S rRNA gene sequences of TRFs 120 and 233 cluster with each other. The 16S rRNA gene sequence of TRF 275 was related to *Deltaproteobacteria*. It was not, however, closely related to the known naphthalene degraders. Rather, this 16S rRNA gene sequence clusters with a phylogenetic group not previously known to mediate naphthalene degradation. Figure 6 displays the phylogenetic tree of the clone corresponding to TRF 215 within *Chlorobi*. It was most closely related to *Prosthecochloris*, a taxon not previously known to be involved in contaminant cycling.

The rapid incorporation of the ^13^C label by the organisms corresponding to TRF 120 and 233 indicates that these organisms are likely responsible for naphthalene ring cleavage (Figure 1B and Figure 4B). While the increase in relative abundance of TRF 275 corresponded to later stages of naphthalene degradation, it does suggest this microorganism can also play a role in naphthalene degradation. TRF 275 represents naphthalene metabolism by an anaerobic organism with a 16S rRNA gene sequence differing from the known anaerobic naphthalene degraders. While the association of the 16S rRNA gene corresponding to TRF 275 with *Desulfomicrobium thermophilum* was low (84% identity), this 16S rRNA gene shared 89% and 88% identity to two clones identified in a petroleum-contaminated aquifer (JQ086867 and JQ086797). *D. thermophilum* has been identified as a prominent sulfate-reducing bacterium inhabiting petroleum reservoirs and oil production facilities [27,28]. The ability, however, of sulfate reducers distantly related to *Desulfomicrobium* to degrade naphthalene or other PAHs has not been reported.

The incorporation of ^13^C by organisms corresponding to the remaining TRFs (215, 120, 233) in the SIP cultures is consistent with the utilization of downstream ^13^C metabolites from ^13^C-naphthalene degradation, produced by the primary naphthalene degraders. Gallagher et al. [29] demonstrated that the organisms responsible for ring cleavage were not the most abundant in the earliest SIP profile. Rather, the microorganisms consuming the downstream ^13^C-metabolites of cleaved aromatic ring products were present in higher relative abundance and increased later in the incubation. 

Our findings regarding the abundance of TRF 215 are also consistent with the phylogenetic affiliation of the corresponding 16S rRNA gene. *Prosthecochloris* is a member of *Chlorobi*, which are obligately phototrophic green sulfur bacteria that are surprisingly common in dark habitats. Its ability to utilize extremely low levels of radiation (0.015 µmol quanta m^−2^ s^−1^) allows *Prosthecochloris* to thrive in deep sediment, hydrothermal vents, and dark bioreactors [30,31,32]. *Prosthecochloris* fixes CO_2_ and can photoheterotrophically assimilate acetate, propionate, peptone, and yeast extract [22]. Given its known metabolism and slow enrichment, the *Prosthecochloris* relative most likely assimilated ^13^C into its DNA by consuming downstream ^13^C intermediate metabolites excreted by primary naphthalene degraders. 

## 4. Conclusions

To our knowledge, all previously studied anaerobic marine naphthalene degraders were enriched from highly contaminated sediments. The naphthalene degrading consortium in this study, in contrast, was obtained from sediment un-impacted by high levels of hydrocarbon pollution. Thus, using SIP allowed for the identification of organisms involved in the natural attenuation of naphthalene in an unimpacted estuary. Microorganisms corresponding to TRFs 120, 233, and 275 demonstrated early and relatively stable enrichment on ^13^C from naphthalene. The close phylogenetic relationship with known anaerobic naphthalene degraders suggests that microbes represented by TRFs 120 and 233 were responsible for initial naphthalene degradation. The geographical distance of our samples from that of NaphS2 and NaphS3 environments in Europe suggests that naphthalene degraders are widely dispersed in sediments, regardless of contamination. The abundance of TRF 275 shares similar enrichment pattern, supporting that this may represent either an additional initial naphthalene degrader or early consumer of naphthalene metabolites. The fact that its relative enrichment peaks at day 14, concurrent with naphthalene consumption, suggests that it is a new anaerobic naphthalene degrader. Further study is necessary to confirm the role of the individual represented by TRF 275 in naphthalene degradation. The *Prosthecochloris* member, corresponding to TRF 215, is also a dominant member of this community. Its presence and ^13^C incorporation in the highly enriched culture suggest the involvement of contaminant-tolerant non-degraders in downstream metabolite carbon cycling.

In petroleum-contaminated environments, naphthalene is only one of many different hydrocarbon structures from mono-aromatic to multi-ringed aromatic hydrocarbons. The naphthalene consortium described here thus occupies a rather narrow and specific metabolic niche, namely, its ability to anaerobically metabolize only naphthalene and 2-methylnaphthalene. We and others have documented the anaerobic degradation of alkanes, monoaromatics, and high molecular weight PAHs under sulfate-reducing conditions by pure and mixed cultures [3,33,34]. For pure cultures of anaerobic alkane degraders only alkanes and related fatty acids served as carbon sources [33,35,36]. These observations underscore the notion that in the anoxic environment a suite of organisms have evolved that are narrow substrate specialists, and thus a community is required for the degradation of hydrocarbons in these habitats. Finally, our SIP study has shown how ^13^C-naphthalene can be expected to move through anaerobic microbial trophic systems. This information can help us better appreciate that the presence and activity of these naphthalene-degrading microorganisms may serve a valuable role to prevent naphthalene from accumulating, despite continual, ubiquitous deposition from point and non-point sources.

## Figures and Tables

**Table 1:** Total measured PAHs in Barnegat Bay sediment compared with Arthur Kill, North Sea, and Etang de Berre sediment, inoculum locations of previous anaerobic naphthalene degradation enrichments. The presence (+) or absence (−) of oil contamination is also indicated.

Water System	Reported PAH Range (μg/kg)	Evidence of Naphthalene Degradation	Oil Contamination?
Barnegat Bay, NJ [10]	Σ_18_PAH = 37–1696	Enriched in this study	−
Mullica River, NJ [11]	Σ_25_PAH = 436–1380	Least disturbed river in North East USA, naphthalene degradation not evaluated	−
Arthur Kill, NY/NJ [3,12]	Σ_26_PAH = 3192–11484	1st anaerobic naphthalene degradation enrichment	+
North Sea, Germany/Denmark [5,13]	Σ_22_PAH = 849–3769	NaphS3	+
Etang de Berre, France [4,14]	Σ_16_PAH = 1595–3359	NaphS2	+

**Table 2:** Sulfate loss during naphthalene degradation. Cultures were amended with 0.5 mM (500 µM) naphthalene in triplicate active cultures and duplicate sterile controls. The stoichiometry of complete naphthalene oxidation to CO_2_ is illustrated.

	Predicted SO_4_^2−^ Loss (mM)	Observed SO_4_^2−^ Loss (mM)
Active Cultures	3.0	3.0 (±1.2)
Sterile Cultures	0	0 (±0)
C_10_H_8_ + 6 H_2_SO_4_ → 10 CO_2_ + 6 H_2_S + 4 H_2_O		

## Supplementary Materials

The following are available online at http://www.mdpi.com/2076-2607/6/3/59/s1, Figure S1: Agarose gel of bacterial 16S rRNA gene amplicons from either ^12^C-naphthalene control or ^13^C-naphthalene SIP incubation.
