# Supplementary material for: Sulfate-Reducing Naphthalene Degraders Are Picky Eaters
Source: Microorganisms. 2018 Jun 25;6(3):59. doi: 10.3390/microorganisms6030059 (PMC6163709; doi:10.3390/microorganisms6030059)
Supplement: Supplementary file 1 [file microorganisms-06-00059-s001.pdf]

Supplemental Material

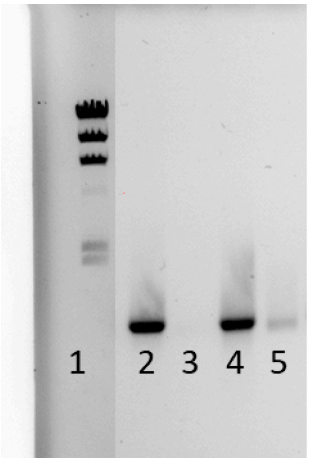

Figure S1. Agarose gel of bacterial 16S rRNA gene amplicons from either ^{12}C-naphthalene control or ^{13}C-naphthalene SIP incubation. 1) lambda DNA; 2) ^{12}C-naphthalene control ^{12}C-DNA 3) ^{12}C-naphthalene control ^{13}C-DNA; 4) SIP incubation day 7 ^{12}C-DNA; 5) SIP incubation day 7 ^{13}C-DNA.
